# DEVELOPMENT OF A BIOMARKER TEST FOR TIGIT IN A DIVERSE COHORT OF HUMAN PANCREATIC CANCER PATIENTS

**DOI:** 10.51894/001c.122822

**Published:** 2024-08-30

**Authors:** Madison George, Julie Clark, Kendyll Gartrelle, Georges Nassif, Kailee Hartway, Daniel Long, Daniel J. Sålas-Escabillas, Allison Wombwell, Hui-Ju Wen, Simone Benitz, Samuel Zwernik, Rupen Shah, Hakmin Park, Philip A. Philip, Gazala Khan, Howard Crawford, David Kwon, Brian Theisen, Nina G. Steele

**Affiliations:** 1 College of Osteopathic Medicine Michigan State University

## 12

### INTRODUCTION

Pancreatic ductal adenocarcinoma (PDAC) has a dismal 12% 5-year survival rate (SEER) due to a lack of early detection biomarkers and resistance to standard therapeutic options (surgery, chemotherapy, radiation). TIGIT, an immune checkpoint receptor, is a marker of T cell exhaustion and plays a key role in the inhibition of anti-tumor immune responses. TIGIT inhibitors are being explored in clinical trials in pancreatic cancer.

### HYPOTHESIS

We hypothesize that targeted anti-TIGIT therapy, in conjunction with other therapies targeting the tumor microenvironment, could reverse the immune suppression characteristic of PDAC.

### METHODS

We performed RNAscope in situ hybridization (ISH) with a probe specific for human TIGIT mRNA (combined with a nuclear counterstain) on 79 tissue samples. The cohort of samples included 8 biopsies (endoscopic-guided fine needle biopsies at time of diagnosis), 66 primary (from surgical resection), and 5 metastatic (liver core biopsies from patients with a primary PDAC diagnosis) formalin-fixed paraffin-embedded (FFPE) tissue sections from patients with histologically confirmed PDAC. The presence of TIGIT probe within the cytoplasm of each cell was determined and quantified. Percent positive cell values were then exported for statistical analysis in R. De-identified clinical metadata was obtained from a cloud-based HIPAA-compliant database. We tested for associations between %TIGIT present and clinical covariates.

### RESULTS

ScRNAseq revealed that TIGIT mRNA is enriched in, but not exclusive to the T/NK cellular compartments in PDAC. Staining analysis showed that TIGIT expression did not differ significantly between racial groups (comparing Black African American (BAA) to non-BAA). The mean percentage of TIGIT positive cells was 64.0%. High expression of TIGIT was associated with clinical stage, where an increase in stage was associated with increasing %TIGIT (p<0.05).

### CONCLUSIONS

The TIGIT biomarker assay can be conducted at time of diagnosis, surgical resection, and metastatic biopsy. If patients’ samples contain high levels of TIGIT expression, these patients may be candidates for anti-TIGIT drug therapy. Considering that TIGIT expression correlates with advancing clinical stage, patients with more advanced staging at diagnosis (stage IIB, III, IV) may especially benefit from anti-TIGIT therapy.

